# Duty of Dentists to Instruct Their Patients Respecting the Care of the Teeth

**Published:** 1867-09

**Authors:** A. Berry


### DUTY OF DENTISTS TO INSTRUCT THEIR PATIENTS RESPECTING THE CARE OF THE TEETH.

BY A. BERRY, D. D. S.

(Read before the Mad River Dental Association, Sept, 3, 1867.)

Mr. President and Fellows of the Association: That there is a great want of knowledge in the community respecting diseases of the teeth and gums, and their proper treatment, is but too evident to every Dentist. Such ignorance is sometimes met with where least expected.

Dr. Brockway in the *Dental Cosmos* for June last gives an extract from an article by Lawrence McKay, M. D., Rochester, N. Y., on the "Gingival Margin as a Diagnostic Sign," published in the last volume of the Transactions of the New York State Medical Society, as follows:

"This margin shows different appearances in different cases; in some it is a mere red line along the edges of the teeth; in others it appears red and congested fully one-eighth of an inch, and even the whole depth of the gums; again, it appears red and spongy, and secretes a pus-like fluid; in other cases the gums appear spongy, and chiseled away from the edges of the teeth, showing their roots in a carious and filthy condition.

"Dentists are in the habit of attributing all these changes to the accumulation of tartar round the roots and along the edges of the teeth, which they dig and scrape off, to the great injury of the patients."

The remarks about Dentists is entirely foreign to the discussion of the subject. Instead of Dr. McKay going out of his way to indulge in wholesale slander of the Dental profession, he could have done better by studying anatomy sufficiently to understand that the gums do not reach the edges of the teeth when these organs are fully developed, only embracing them at their necks.

It is fortunate that injury from the removal of salivary calculus from the teeth by Dentists, exists only in the imagination of Dr. McKay. Perhaps he agrees in opinion with a vender of dentifrice, who said to his wondering street auditory, "Tartar is nothing but bread and butter that got on your teeth when you were little," and advised its removal with his tooth-powder.

If the public were aware of the importance of keeping the teeth free from the deposits of tartar, such egregious nonsense as that its removal by Dentists is done "to the great injury of their patients," would not probably be disseminated by the New York State Medical Society.

As there are no books in general use containing information as to the diseases of the teeth and gums, and the care they require, it is important that Dentists, as conservators of the public weal, do what they may to diffuse knowledge on these subjects; which may be done orally, (not forgetting to state the fact that when the gums are healthy, teeth that are kept clean seldom decay,) and by the distribution of small treatises, such as Dr. Watt's prize Essay, or the smaller *Dental Luminary*, published by the Cincinnati Dental Association.

The sixth year molars afford an example. Generally supposed to belong to the temporary set, they are usually neglected when carious, until odontalgia causes a resort to the Dentist; when had the parents been well informed those having proper regard for the welfare of their children would have had these teeth filled at an early stage of decay.

Again, how often do we see instances of neglect to keep the teeth clean and the gums healthy, causing extensive caries of the teeth, and frequently absorption of the alveoli, and loss of sound teeth.

Another important consideration is, that the fetor from badly diseased teeth and gums is not only annoying to others, but taints the air passing into the lungs, while the pus exuding in many cases from the gums is mixed with the food and finds its way into the stomach: the fetor and pus thus seriously injuring the health of the patient.

